# Supplementary material for: A matched case-control analysis of autonomous vs human-driven vehicle accidents
Source: Nat Commun. 2024 Jun 18;15:4931. doi: 10.1038/s41467-024-48526-4 (PMC11189485; doi:10.1038/s41467-024-48526-4)
Supplement: Supplementary file 1 — Supplementary Information [file 41467_2024_48526_MOESM1_ESM.pdf]

Supplementary Information for

A Matched Case-Control Analysis of Autonomous vs Human-Driven Vehicle Accidents

Mohamed Abdel-Aty[1]
Professor
m.aty@ucf.edu

Shengxuan Ding[1]*
Graduate Research Assistant
shengxuan.ding@ucf.edu

1. Department of Civil, Environmental and Construction Engineering, University of Central Florida, 12800 Pegasus Dr #211, Orlando, FL 32816, USA

*Corresponding author

The PDF file includes:

Supplementary Methods

Supplementary Table

Supplementary Reference

Supplementary Methods

The prior probability of accidents (i.e., the frequency of accidents per distance traveled) for AVs and HDVs is also crucial to this study. To delve deeper into the accident likelihood under non-accident conditions for AVs, we sourced data on autonomous vehicle miles traveled (AVMT), disengagement accidents, and accident occurrences from the publicly available California DMV AV accident and disengagement reports[1]. We calculated the disengagement rate per 1,000 miles and the accident rate per 1,000 miles.

Supplementary Table. 1 Number of AV AVMT, disengagement and Accidents

Year	AVMT	Disengagement	Disengagement/1000 mi	Accident	Accident /1000 mi
2017	483786	11281	23.32	22	0.05
2018	1971474	66049	33.5	44	0.02
2019	2636198	1972	0.75	67	0.03
2020	1565353	335	0.21	20	0.01
2021	4051850	8216	0.2	117	0.03
2022	5964804	2376	0.04	150	0.03

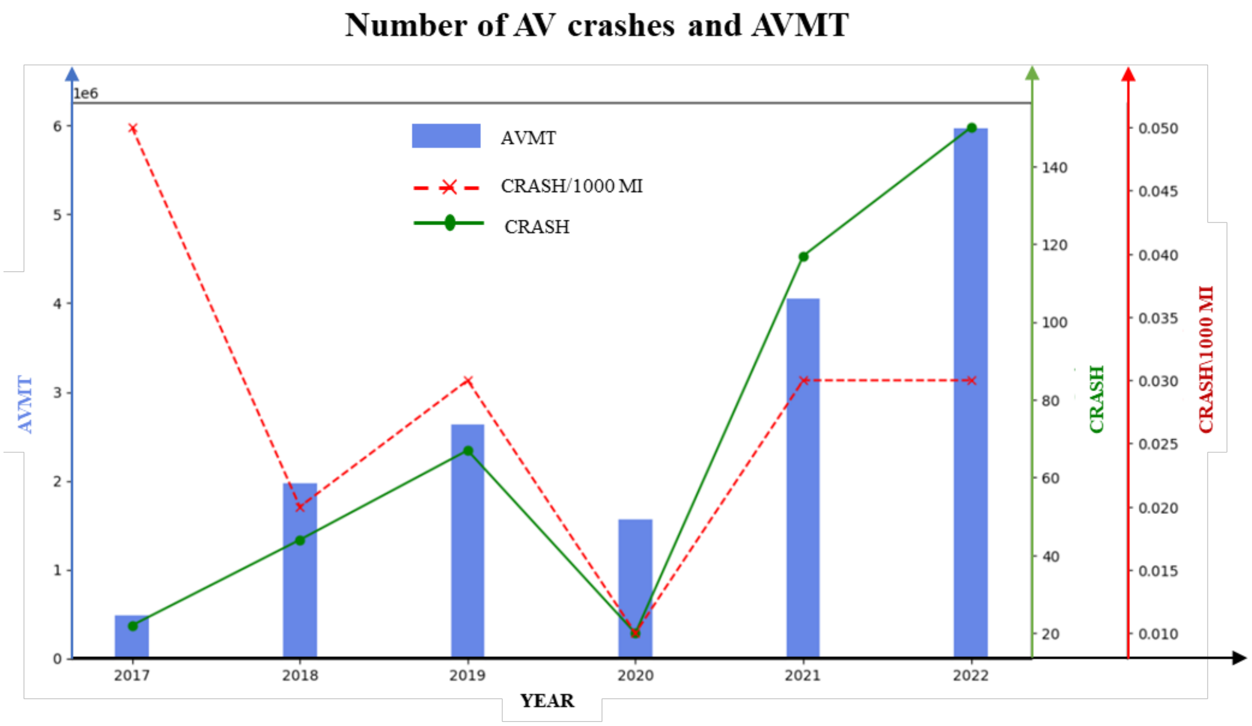

Supplementary Figure. 1 Number of AV AVMT and Accidents

For the HDV group, we gathered data on Motor Vehicle Miles of Travel (MVMT) from the SWITRS Annual Report of Fatal and Injury Motor Vehicle Traffic Collisions[2]. This information allowed us to compute the Accident rate per 1 million miles for HDVs.

Supplementary Table. 2 Number of HDV MVMT and Accidents

Year	MVMT	Accident	Accident /1 million mi
2010	327770000000	161094	0.05
2011	325032000000	159115	0.05
2012	326547000000	159696	0.05
2013	329174000000	156909	0.05
2014	334664000000	162742	0.05
2015	339843000000	178669	0.05
2016	342853000000	195347	0.06
2017	344304000000	193564	0.06
2018	347194000000	191971	0.06
2019	351151000000	187211	0.05

*Data after 2019 is Not yet available

The method for calculating accident rates takes into consideration vehicle exposure by including AADT[3]. The accident rates at segments can be determined using the equation below:

$$R_{section} = \frac{A * 1,000,000}{365 * T * V_{section} * L}$$

Where

R_{section} is the accident rate for the section.

A is the number of reported accidents during the time period.

T is the number of years considered.

$V_{section}$ is AADT or annual average daily traffic, vehicles per day.

L is the length of section, miles.

For intersections, we use the total entering AADT as the variable V. The accident rates at intersections can be determined using the equation below[4]:

$$R_{spot} = \frac{A * 1,000,000}{365 * T * V_{spot}}$$

Where

R_{spot} is the accident rate for the spot.

A is the number of reported accidents during the time period.

T is the number of years considered.

V_{spot} is AADT or annual average daily traffic ($V_{spot} = (AADT_{minor\ roads} + AADT_{major\ roads})$), vehicles per day.

The accident rate and number of accident comparison by road type is shown as the chart below. Accident rates are classified into four categories: Highways, Intersections, Rural Roads, and Streets. HDVs show a higher incidence of accidents on highways, intersections, and streets, particularly on highways. For rural roads, HDVs and AVs exhibit similar accident rates. Across all road types, HDVs consistently record significantly higher accident figures than AVs.

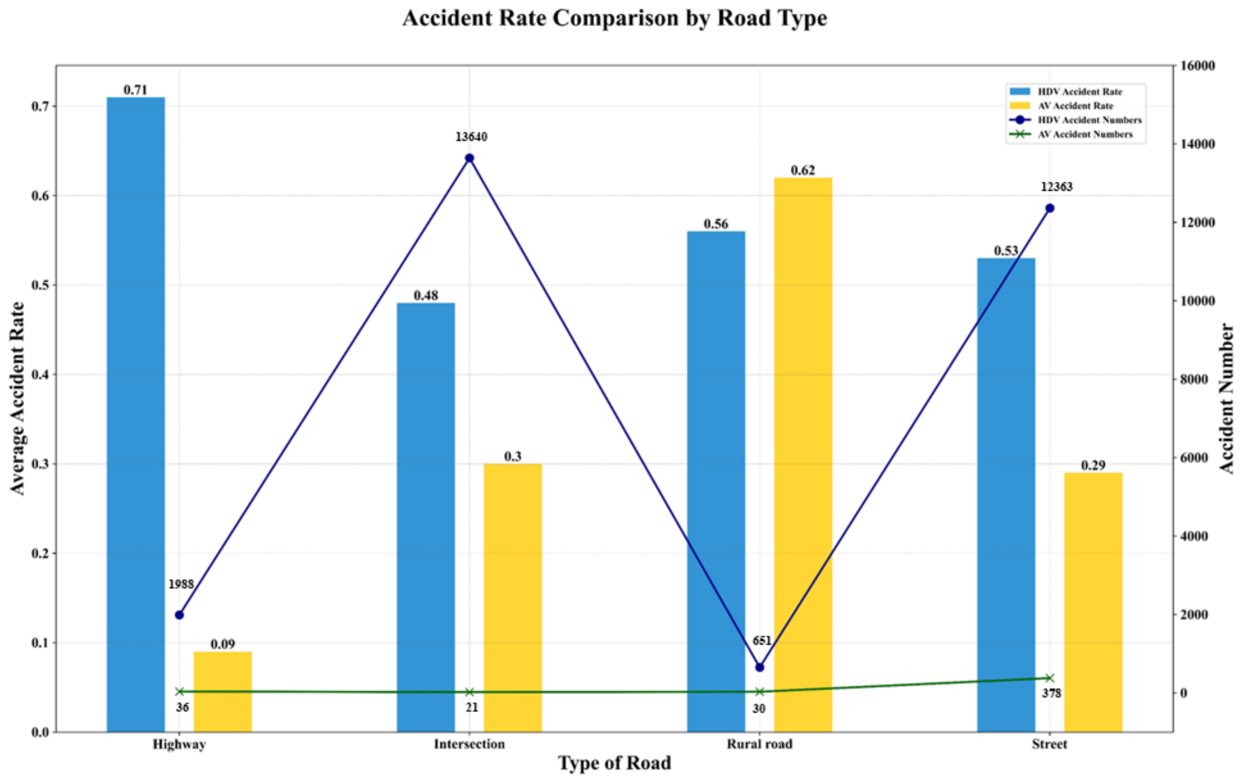

Supplementary Figure. 2 Accident Rate comparison by Road type

This distribution under different scenarios such as weather, is consistent with the results of matched case-control study conducted. The accident rate is consistent with the result of matched case-control that the odds of an AV (ADS) accident occurring in rainy weather are 0.336 times, as the chart shown below. This indicates a lower likelihood of an AV accident in rainy weather compared to an HDV accident.

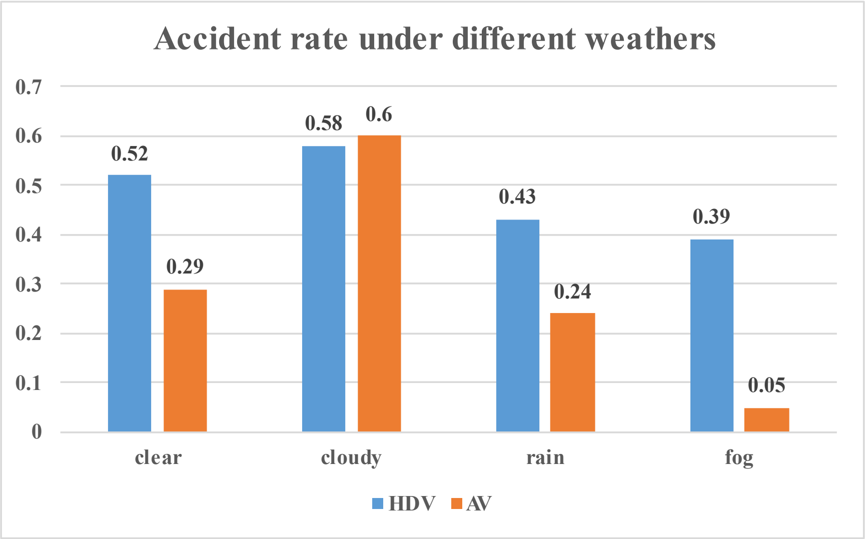

Supplementary Figure. 3 Accident Rate under different weathers

Direct comparison between AV and HDV accidents is still not viable as the difference in exposure and number of vehicles of both types are extremely unbalanced.

Supplementary Table

Supplementary Table 3 Summary statistics for the variables included in the generated datasets.

Variable type	Variable description	AV (ADS+ADAS) (2100)		ADS (For matched case control study) (548)		HDV (35,133)	
		Mean	Standard deviation	Mean	Standard deviation	Mean	Standard Deviation
**Road and environment**							
Weather	Clear condition (1 if weather is clear, 0 otherwise)	0.7275	0.2404	0.8719	0.3404	0.8343	0.3718
	Cloudy condition (1 if weather is cloudy, 0 otherwise)	0.1515	0.1042	0.0898	0.2642	0.1105	0.2135
	Rain condition (1 if weather is rain, 0 otherwise)	0.1124	0.1952	0.0292	0.1252	0.0519	0.2218
	Fog condition (1 if weather is fog, 0 otherwise)	0.0086	0.0189	0.0091	0.1189	0.0033	0.1574
Road Condition	Normal road condition (1 if there are no unusual conditions like construction, maintenance, or obstruction, 0 otherwise)	0.9353	0.1171	0.9727	0.2171	0.9863	0.2160
	Traffic event/work zone (1 if there are traffic event/work zone, 0 otherwise)	0.0547	0.1633	0.0182	0.0633	0.0126	0.2114
	Temporary reduced Roadway Width (1 if road condition is reduced roadway width, 1 otherwise)	0.0100	0.0485	0.0091	0.0556	0.0011	0.0329
Road Surface	Dry surface condition (1 if surface is dry, 0 otherwise)	0.8665	0.2952	0.8810	0.2884	0.8854	0.3185
	Wet surface condition (1 if surface is wet, 0 otherwise)	0.1198	0.2781	0.0867	0.2781	0.1034	0.2323
	Snow/slush/ice condition (1 if surface is covered with snow/slush/ice, 0 otherwise)	0.0137	0.1427	0.0323	0.2427	0.0112	0.1321
Lighting	Daylight condition (1 if daylight, 0 otherwise)	0.7082	0.3523	0.7556	0.4023	0.6669	0.4755
	Dawn/dusk condition (1 if it is dawn/dusk, 0 otherwise)	0.0347	0.2552	0.0255	0.1276	0.0489	0.1484
	Dark– lighting condition (1 if it is dark–lit, 0 otherwise)	0.2081	0.2265	0.2153	0.3265	0.2753	0.3520
	Dark– not lighting condition (1 if it is dark– not lit), 0 otherwise)	0.0490	0.1251	0.0036	0.0351	0.0089	0.1880
**Pre-accident conditions**							
Associated Factor	Inattention (1 if driver is distracted, 0 otherwise)	0.0176	0.0952	0.0091	0.2191	0.1975	0.1981
	Entering/Leaving Ramp (1 if driver is Entering/Leaving Ramp, 0 otherwise)	0.0157	0.1420	0.0146	0.01386	0.021	0.1433
	Defective Vehicle Equipment (1 if Vehicle Equipment is defective, 0 otherwise)	0.0229	0.0685	0.0292	0.1292	0.0012	0.1305
	No dangerous driver behavior (1 if Vehicle is no dangerous driver behavior, 0 otherwise)	0.9438	0.2748	0.9471	0.2671	0.7803	0.4234
Pre-accident	Stopped (1 if pre-accident movement is stopped, 0 otherwise)	0.1781	0.2523	0.1651	0.2523	0.0016	0.2399

moveme nt	Proceeding straight (1 if pre-accident movement is proceeding straight, 0 otherwise)	0.5554	0.4758	0.458	0.4758	0.5807	0.4935
	Run-off Road (1 if pre-accident movement is Run-off Road, 0 otherwise)	0.0495	0.1296	0.0182	0.1236	0.0171	0.1297
	Turn (1 if pre-accident movement is turning left or right, 0 otherwise)	0.0876	0.2723	0.1077	0.3272	0.1049	0.2065
	Backing (1 if pre-accident movement is backing, 0 otherwise)	0.0152	0.2007	0.0191	0.4252	0.0282	0.1655
	Changing lanes (1 if pre-accident movement is changing lanes, 0 otherwise)	0.0519	0.2312	0.0949	0.1312	0.0661	0.3485
	Entering traffic lane (1 if pre-accident movement is entering traffic lane, 0 otherwise)	0.0283	0.1404	0.0903	0.3391	0.0303	0.1715
	Into opposing lane (1 if pre-accident movement is into opposing lane, 0 otherwise)	0.0114	0.1434	0.0146	0.1034	0.1634	0.3697
	Parked (1 if pre-accident movement is parked, 0 otherwise)	0.0138	0.3951	0.0201	0.2404	0.0015	0.1385
	Travelling wrong way (1 if pre-accident movement is travelling wrong way, 0 otherwise)	0.0088	0.3381	0.0120	0.1381	0.0062	0.2787
Accident Participa tion	Pedestrian or bicycle (1 if accident with pedestrian or bicycle, 0 otherwise)	0.0319	0.0952	0.0547	0.4952	0.151	0.3404
	Other participants (1 if accident with other participants, 0 otherwise)	0.1652	0.3341	0.0584	0.2341	0.2227	0.2161
	Vehicle (1 if accident with another vehicle, 0 otherwise)	0.8029	0.4789	0.8869	0.4789	0.6263	0.4838
**Accident type**							
	Head-on (1 if accident type is Head-on, 0 otherwise)	0.3271	0.4534	0.2391	0.3391	0.3268	0.3857
	Sideswipe (1 if accident type is Sideswipe, 0 otherwise)	0.2019	0.2076	0.2099	0.2483	0.1460	0.2489
	Rear-end (1 if accident type is Rear-end, 0 otherwise)	0.3919	0.3921	0.4818	0.4725	0.4533	0.4756
	Broadside (1 if the front of one vehicle slams into the side of another vehicle, 0 otherwise)	0.0079	0.1735	0.0693	0.1335	0.0739	0.1312
**Accident outcomes**							
Severity	No-injury (1 if injury severity outcome is no-injury, 0 otherwise)	0.8971	0.3114	0.6570	0.4114	0.6876	0.4771
	Minor (1 if injury severity outcome is minor, 0 otherwise)	0.0410	0.1604	0.2609	0.3302	0.247	0.3467
	Moderate (1 if injury severity outcome is moderate, 0 otherwise)	0.0399	0.0302	0.0803	0.2604	0.0565	0.1484
	Fatal (1 if injury severity outcome is fatal, 0 otherwise)	0.0221	0.1579	0.0018	0.1579	0.0089	0.1945

*Note:

The mean serves as an indication of the percentage of data that have the value 1.

The standard deviation measures the variability of the binary data. A lower standard deviation suggests results near the mean, indicating a dominance of either 0 or 1, while a higher standard deviation indicates a more balanced distribution of 0s and 1s.

Supplementary Table 4 Description and explanation for the variables in this paper

Variable type	Variable Value	Variable Description
**Road and environment**		
Road Condition	Normal road conditions (1 if there are no unusual conditions like construction, maintenance, or obstruction, 0 otherwise)	Self-explanatory.
	Traffic event/work zone (1 if there are traffic event/work zone, 0 otherwise)	If a prior traffic event such as traffic accidents, disabled vehicles, and spilled cargo that adversely affected normal traffic operations separate from the reported incident or work zone.
	Temporary reduced Roadway Width (1 if road condition is reduced roadway width, 1 otherwise)	For example, snow drifts, dirt slides, construction zones, etc.
**Pre-accident conditions**		
Associated Factor	Inattention (1 if driver is distracted, 0 otherwise)	It was an associated factor in the collision. For example, adjusting radio, lighting a cigarette, conversing with passengers, etc.
	Entering/Leaving Ramp (1 if driver is Entering/Leaving Ramp, 0 otherwise)	It includes collisions occurring on the ramp. For example, a driver starts to enter an on-ramp at an intersection and collides with another vehicle.
	Defective Vehicle Equipment (1 if Vehicle Equipment is defective, 0 otherwise)	For example, brakes, headlights, tread depth, etc.
	Uninvolved Vehicle (1 if Vehicle is uninvolved, 0 otherwise)	The involved party which claims that another vehicle contributed to the collision.
	No dangerous driver behavior (1 if there is no dangerous driver behavior, 0 otherwise)	Self-explanatory.
Pre-accident movement	Stopped (1 if pre-accident movement is stopped, 0 otherwise)	A vehicle not moving but, on the roadway, (excludes shoulder). A stalled, disabled, or abandoned vehicle on a roadway is considered stopped.
	Proceeding straight (1 if pre-accident movement is proceeding straight, 0 otherwise)	A vehicle proceeding straight ahead. A vehicle following the curve of a roadway is coded proceeding straight.
	Run-off Road (1 if pre-accident movement is Run-off Road, 0 otherwise)	If the motor vehicle left the road (includes paved or unpaved shoulder) prior to the collision and before evasive action began. This includes vehicles which would have left the road had their movement not been halted by colliding with protective barriers such as guardrails, concrete walls, or median barriers which are generally placed adjacent to, but outside the road.
	Turn (1 if pre-accident movement is turning left or right, 0 otherwise)	A vehicle making a turn at an intersection or into a private drive, or a vehicle stopped within an intersection preparing to turn. This includes an illegal turning movement, such as a turn when prohibited or when out of position. This excludes any lane change or turning movement to pass other vehicles.
	Backing (1 if pre-accident movement is backing, 0 otherwise)	A vehicle backing up, except when associated with parking.
	Slowing (1 if pre-accident movement is Slowing, 0 otherwise)	A vehicle in the process of slowing or stopping its forward movement. Speed is not a factor in determining whether this movement applies.

	Changing lanes (1 if pre-accident movement is changing lanes, 0 otherwise)	A vehicle making a lane change on a roadway divided into two or more clearly marked lanes for traffic in one direction.
	Entering traffic lane (1 if pre-accident movement is entering traffic lane, 0 otherwise)	A vehicle entering a designated lane of traffic from a shoulder, median, parking strip, alley, or private drive. Usually this is a forward movement, but it may include a backing movement associated with leaving a parked position.
	Into opposing lane (1 if pre-accident movement is into opposing lane, 0 otherwise)	A vehicle making an involuntary or unplanned movement into an opposing lane of traffic on a two-way road.
	Parked (1 if pre-accident movement is parked, 0 otherwise)	A vehicle not moving and outside of a traffic lane. This includes a vehicle stopped on a shoulder or in another area where parking is designated or permitted or a motor vehicle parked illegally but otherwise outside a traffic lane, such as blocking a driveway, beside a fire hydrant, or in a loading zone.
	Travelling wrong way (1 if pre-accident movement is travelling wrong way, 0 otherwise)	A vehicle proceeding in the opposite direction of traffic.
Accident Participation	Pedestrian (1 if accident with pedestrian, 0 otherwise)	A collision involves a bicycle or a motor vehicle in-transport and a pedestrian. Includes a person in or operating a coaster wagon, scooter, sled, skateboard, wheelchair, motorized wheelchair, or "electric personal assistive mobility device".
	Other participants (1 if accident with other participants, 0 otherwise)	A collision involves a motor vehicle in-transport which comes in contact with another motor vehicle upon the same roadway or upon roadways within an intersection. Falling loads, detached trailers, etc., are considered part of the original motor vehicle.
	Vehicle (1 if accident with another vehicle, 0 otherwise)	A collision involves a motor vehicle in-transport which leaves the roadway and collides with another motor vehicle in-transport on another roadway. For example, a vehicle crosses a median strip and collides with a vehicle on an opposing roadway.
**Accident type**		
	Head-on (1 if accident type is Head-on, 0 otherwise)	Two motor vehicles, approaching from opposite directions, make direct contact. For example, the front of one vehicle collides with the front of another. Or prior to impact, one vehicle skids sideways, causing the side of the skidding vehicle to collide with the front of the other.
	Sideswipe (1 if accident type is Sideswipe, 0 otherwise)	One motor vehicle strikes the side of another with a glancing blow. For example, two vehicles are proceeding in the same direction or from opposite directions, and the side of one vehicle strikes the side of the other.
	Rear-end (1 if accident type is Rear-end, 0 otherwise)	Two motor vehicles, traveling in the same direction, make direct contact. For example, the front of one vehicle strikes the rear of another vehicle, or Vehicle #1 approaches Vehicle #2 from the rear and skids sideways during a braking action, causing the side of Vehicle #1 to strike the rear of Vehicle #2.
	Broadside (1 if the front of one vehicle slams into the side of another vehicle, 0 otherwise)	One motor vehicle strikes another vehicle at an angle greater than that of a sideswipe.
	Hit-object (1 if accident type is Hit-object, 0 otherwise)	A motor vehicle strikes a fixed object or other object.
**Accident outcomes**		
Severity	No-injury (1 if injury severity outcome is no-injury, 0 otherwise)	Self-explanatory.

	Minor (1 if injury severity outcome is minor, 0 otherwise)	This contains other visible injuries and complaint of pain. An injury, other than a fatal or severe injury, which is evident to observers at the scene of the collision. Other visible injuries include: (1) Bruises, discoloration, or swelling. (2) Minor lacerations or abrasions. (3) Minor burns. This classification could also contain authentic internal, other non-visible injuries, and fraudulent claims of injury. “Complaint of Pain” includes: (1) Persons who seem dazed, confused, or incoherent (unless such behavior can be attributed to intoxication, extreme age, illness, or mental infirmities). (2) Persons who are limping, or complaining of pain or nausea, but do not have visible injuries. (3) Any person who may have been unconscious, as a result of the collision, although it appears he/she has recovered. (4) Persons who say they want to be listed as injured but do not appear to be so.
	Moderate (1 if injury severity outcome is moderate, 0 otherwise)	An injury, other than a fatal injury, that includes the following: (1) Broken or fractured bones. (2) Dislocated or distorted limbs. (3) Severe lacerations. (4) Skull, spinal, chest or abdominal injuries that go beyond “Other Visible Injuries.” (5) Unconsciousness at or when taken from the collision scene. (6) Severe burns.
	Fatal (1 if injury severity outcome is fatal, 0 otherwise)	Death as a result of injuries sustained in a collision, or an injury resulting in death within 30 days of the collision.

Supplementary References

1 DMV, C. *DISENGAGEMENT REPORTS*, <https://www.dmv.ca.gov/portal/vehicle-industry-services/autonomous-vehicles/disengagement-reports/> (2024).

2 System, S.-S. I. T. R. <https://www.chp.ca.gov/programs-services/services-information/switrs-internet-statewide-integrated-traffic-records-system/> (2024).

3 Wang, T., Gan, A. & Alluri, P. Estimating annual average daily traffic for local roads for highway safety analysis. *Transportation research record* **2398**, 60-66 (2013).

4 Wu, J. & Xu, H. Annual average daily traffic prediction model for minor roads at intersections. *Journal of Transportation Engineering, Part A: Systems* **145**, 04019041 (2019).
